# MR imaging of primary benign cardiac tumors in the pediatric population

**DOI:** 10.1016/j.heliyon.2023.e19932

**Published:** 2023-09-12

**Authors:** Maria Cristina Inserra, Maria Teresa Cannizzaro, Giulia Passaniti, Antonio Celona, Aurelio Secinaro, Davide Curione, Tommaso D'Angelo, Orazio Garretto, Placido Romeo

**Affiliations:** a UOSD Radiologia 2 CAST, A.O.U. Policlinico “G. Rodolico - San Marco”, Catania, Italy; b Division of Cardiology, A.O.U. Policlinico “G. Rodolico - San Marco”, Catania, Italy; c UOC Radiodiagnostica, San Vincenzo Hospital, Provincial Health Agency of Messina, Taormina, Italy; d Advanced Cardiothoracic Imaging, Bambino Gesù Children's Hospital, IRCCS, Rome, Italy; e Diagnostic and Interventional Radiology Unit, BIOMORF Department, University Hospital “Policlinico G. Martino”, Messina Italy; f Department of Radiology and Nuclear Medicine, Erasmus MC, Rotterdam, the Netherlands; g Radiology Department of AO “San Marco”, A.O.U. Policlinico “G. Rodolico - San Marco”, Catania, Italy

## Abstract

Primary cardiac tumors are rare in all ages, especially in children, with a reported prevalence range of 0.0017–0.28% in autopsy series. Due to their rarity, the diagnostic and therapeutic pathways reserved to them are usually described by single case reports, leading to the point where a common diagnostic protocol is imperative to obtain a differential diagnosis. The first diagnostic approach is done with transthoracic echocardiogram (TTE), due to its wide availability, low cost, absence of ionizing radiations and non-invasiveness. Several tumors are discovered incidentally and, in many cases, TTE is helpful to determine location, size and anatomical features, playing a key role in the differential diagnosis.

In the last few years, cardiac magnetic resonance imaging (CMR) has had an increased role in the diagnostic pathway of pediatric cardiac masses, due to its high accuracy in characterizing mass tissue properties (especially for soft tissue), and in detecting tumor size, extent, pericardial/pleural effusion, leading to the correct diagnosis, treatment and follow-up. Therefore, nowadays, several consensus statements consider CMR as a leading imaging technique, thanks to its non-invasive tissue characterization, without the use of ionizing radiation, in an unrestricted field of view.

As suggested by the most recent literature, the pediatric protocol is not so different from the adult one, adapted to the size and cardiac frequency of the patient, sometimes requiring special conditions such as free-breathing sequences and/or sedation or general anesthesia in non-cooperating patients.

## 1 Introduction

Primary cardiac tumors are rare in all ages, especially in children, with a reported prevalence range of 0.0017–0.28% in autopsy series [1], and 0.15% prevalence in echocardiographic studies [2].

Secondary cardiac tumors are 20 times more common and they are usually the result of remote spreading from distant primary neoplasms, of direct extension from nearby thoracic and/or mediastinal malignancies, or of propagation of thrombo-neoplastic emboli from other body compartments via the caval system [3].

More than 90% of primary cardiac tumors in children are benign, while the other 10% is represented by malignant neoplasms. The oncological classification is based on the pathology of the tumor and it does not take into consideration all the symptoms that the tumor can cause. Commonly, clinical features triggered by the neoplasm display the location, size and extent of the tumor.

Tumors can be totally asymptomatic or clinical manifestations in children can include several signs and symptoms [4]. Cardiac symptoms can be due to obstruction, in particular inflow and outflow tract obstruction that can disrupt normal valvular function, leading to syncope and sudden death [5]. Moreover, the heart tumor can interfere with the normal electrical conduction system of the heart, causing arrhythmias, atrio-ventricular blocks, and eventually ventricular fibrillation and cardiac arrest [6]. Embolization tendency depends on many factors such as tumor origin (intracavitary or intramural mass), histology and endocavitary surface: masses with a friable surface have a higher chance of embolization. Depending on the location of the mass, it can determine pulmonary or systemic embolism [7]. Additionally, patients can display systemic manifestations, such as fever, weight loss, fatigue, muscular aches, cough [8].

Due to the widespread variety of symptoms, diagnosing a cardiac tumor can be challenging.

Transthoracic echocardiogram (TTE) is the first line diagnostic tool when a cardiac mass is suspected, due to its wide availability, low cost, absence of ionizing radiations and non-invasiveness. Several tumors are discovered incidentally and, in many cases, TTE is accurate in determining location, size and anatomical features, thus TTE has a cornerstone role in the detection of tiny (5–10 mm) and mobile lesions.

Although its well-established usefulness, TTE carries numerous limitations, such as operator's dependence, restricted field of view and restricted image detection in case of inadequate acoustic windows. These limitations can be partially overcome with the use of transesophageal echocardiogram (TOE), that is nonetheless still an invasive procedure.

In the last few years, CMR has had an increased role in the diagnostic pathway of cardiac pediatric masses, due to its high accuracy in detecting tumor size, extent and in characterizing the mass tissue [9]. Therefore, nowadays, several consensus statements consider CMR as a leading imaging technique, as it has better tissue characterization, without an unrestricted field of view and without the use of ionizing radiation [10,11].

Some limitations of MRI include long acquisition times (at least around 40–50 min), with ECG-gated technique, leading to consequent artifacts in case of arrhythmias; also, small mobile masses may not be adequately visualized; moreover, the need for breath-holding requires the patient to be compliant, meaning that children under 7 years old of age often necessitate sedation or general anesthesia [12,13].

Cardiac tomography (CT) can be used as a second-line technique to assess cardiac tumors, because of its not-negligeable limitations, such as exposure to ionizing radiation and iodinate contrast with lower temporal resolution, compared to TTE or CMR [14].

Its main use would be to assess for the presence of calcifications, which can help in making a diagnosis, and the relation of the mass with the coronary arteries. Nevertheless, CT also has tissue characterization capabilities, particularly with more recent technology as dual energy. This technique has well known benefits in coronary and cardiovascular imaging, eliminating the need for non-contrast exams, which can be reconstructed from contrast-enhanced acquisitions, increasing contrast, reducing noise and beam-hardening artifacts, enhancing perfusion and late enhancement scans, at the cost of an increased radiation dose [15,16]. However, it is still mainly investigational in cardiac masses, usually focusing on the differentiation between tumors and thrombi [17].

## 2 Classification

The WHO updated the classification of Tumors of the Heart in 2021 [18]. This classification divides cardiac masses mainly in two categories as primary cardiac tumors and secondary cardiac tumors.

Based on their clinical and pathological features, primary heart tumors are divided into benign, malignant tumors.

Benign cardiac tumors include Rhabdomyoma, Fibroma, Myxoma, Papillary fibroelastoma, various form of Hemangioma, of Lipoma, of Hamartoma and Cystic tumor of atrioventricular node.

Germ cells tumors (Teratomas) were included in the WHO 2015 classification of Heart Tumors, but they are not integrated in the WHO 2021 edition since myocardial germ cells tumors are extremely infrequent and the vast majority develops from the pericardium.

Instead, malignant tumors are very rare and the fifth edition of the WHO classification distinguishes Sarcomas, namely Angiosarcoma, Leiomyosarcoma and Undifferentiated pleomorphic sarcoma, and Hematolymphoid tumors group including Diffuse and Fibrin-associated large B-cell Lymphoma [18].

Secondary cardiac tumors are more common. Commonly, tumors metastasize to the heart through the mediastinal lymphatics, resulting in epicardial deposition, acute and chronic pericarditis and eventually, cardiac tamponade [19]. Other sources include ovarian, thyroid, thymic, kidney, leukemia, lymphoma and esophageal cancer, in various stages.

A classification of cardiac tumors can be found in Table 1.

**Table 1:** Classification of cardiac tumors.

*Benign*	*Malignant*
Rhabdomyoma	Metastasis
Fibroma	Sarcoma - Angiosarcoma - Leiomyosarcoma - Pleomorphic sarcoma
Myxoma	
Teratoma	
Hemangioma	Lymphoma
Papillary fibroelastoma	
Lipoma	***Tumor-Like***
Hamartoma	Thrombus

Cardiac tumors may occur in any part of the heart and could develop in any chambers, as each cardiac tumor may be more frequent in one of them. Usually, myxomas are found mainly in the left atrium, lipomas in the right atrium or in the left ventricle, and fibromas and rhabdomyomas are frequently discovered in the ventricles. Also, angiosarcomas generally arise in the right atrium, whereas undifferentiated pleomorphic sarcomas occur in the left atrium.

## 3 Cmr technique

CMR is a very accurate technique for identifying and discriminating a suspected cardiac mass.

It clearly delineates the size, the location, the tissue properties (especially for soft tissue) and the presence of pericardial/pleural effusion, leading diagnosis, treatment and follow-up.

As underlined by some authors, it is an excellent tool to depict and define the mass, in order to plan the surgical approach (Class I, Level of Evidence B) [20].

As suggested by several scientists [21] a multiparametric protocol is necessary to evaluate signal components and morphological aspects, with a diagnostic accuracy of 0.92 (AUC) in order to discriminate malignant vs benign masses.

Researchers [22] suggested a core protocol for imaging cardiac masses, underlining exigency for tailored adjustments according to the mass under examination.

The exam should start with a localizer sequence covering the entire thorax (usually axial bright-blood balanced steady-state free precession [b-SSFP] or black-blood single-shot turbo spin echo [TSE] [23]). Then, the main sequences that should always be performed include [24].

− -cine b-SSFP through conventional and mass-customized planes, which must be repeated for all subsequent imaging, in order to confirm the site, size, extension and hemodynamic impact of the mass such as its mobility, attachment points, valvular regurgitation and/or in/outflow tract obstruction; b-SSFP images are less important for tissue characterization.

When valvular implication is recognized, phase-contrast sequences might be achieved in order to estimate and quantify the hemodynamic involvement [25].

For complete tissue characterization, black-blood images are acquired before contrast administration, typically using a double inversion recovery TSE sequence. This sequence employs an initial non-slice selective 180° inversion pulse to null the blood followed by a slice-selective 180° pulse to restore the signal in the slice of interest. A third slice-selective 180° inversion pulse can be added to obtain fat saturation, making it a triple inversion recovery sequence [26].

Both T1-weighted and T2-weighted with and without fat saturation must then be performed in order to identify fat, hemorrhage or increased water component [22,27].

Performing additional tissue characterization sequences with parametric imaging (mapping) is an increasing trend, and recent consensuses encourage the use to improve experience and future reliability. Novel T1 and T2 mapping can provide quantitative information on the mass. Currently, they are not routinely considered to make a diagnosis, but they have shown to be of help in some instances [28] and may have a more direct impact in the future with more robust data and widespread use.

− First pass perfusion is carried out using a T1-weighted inversion recovery gradient-echo (GRE), hybrid GRE-echo planar imaging (EPI) or SSFP sequence during intravenous infusion of 0.1 mmol/kg of in order to evaluate the lesion vascularization and reveal a hypervascular tumor [29]. High signal is in fact expected in vascular tumors (such as hemangioma, angiosarcoma), while it must be absent or minimal to diagnose other entities (such as rhabdomyoma or fibroma); heterogenous contrast enhancement can be related to differences in the tumor's vascularization due to the existence of necrosis [22].

Early gadolinium enhancement (EGE) can optionally be acquired 2–3 min after contrast administration and is mainly used in the setting of suspected thrombus.

Black-blood T1-weighted images obtained post-contrast are helpful and can suggest malignancy when conspicuous contrast enhancement is shown. However, in the pediatric setting, they are not mandatory, as recently described [13,30].

− Late gadolinium enhancement (LGE) sequences are inversion recovery prepared T1-weighted GRE sequences performed 8–10 min after contrast injection. The inversion recovery pulse is used to null the signal of normal myocardium with the aim to optimize regions of gadolinium accumulation. A correct inversion time (TI) selection is imperative for standard sequences and is chosen by previously applying a TI-scout or Look-Locker slice, while it is less relevant if Phase-sensitive inversion recovery techniques are used.

Additional sequences may include 3D ECG-synchronized sequences such as 3D b-SSFP WHOLE HEART (3D WH) to assess anatomy and, if possible, the relation with the coronaries arteries [31].

The CMR protocol is summarized in Table 2 and the most common MRI features of pediatric cardiac masses can be found in Table 3.

**Table 2:** CMR protocol.

*Sequences*	*Purpose*
*Cine b-SSP*	tumor location, size and extent hemodynamic behavior (e.g. mobility) and impact (e.g. valves)
*Precontrast BB TSE T1w*	tumor signal with fat detection
*Precontrast BB TSE T2w*	tumor signal with water detection
*Perfusion*	tumor vascularization
*LGE*	tumor fibrosis
***Additional***	
*Postcontrast BB TSE T1w*	tumor enhancement
*EGE*	thrombus differentiation
*Mapping*	tumor quantitative parameters
*Phase-contrast*	quantitative valve impact
*3D*	tumor anatomy and relation with the coronary arteries

**Table 3:** MRI features of primary benign pediatric masses.

Tumor type	b-SSFP	T1W	T2W	FPP	LGE
Rhabdomyoma	Iso/hypo	iso	Variable	Weak or absent	Iso/hypo
Fibroma	Hypo/iso	Hypo/iso	Variable	Absent uptake	Avid and progressive with or without dark center
Inflammatory Myofibroblastic Tumor	Mild hyper	Variable	Variable	Absent uptake	Variable
Cystic Tumor of AV Node	Hyper/iso	iso	hyper	Variable	Variable
Hemangioma	Hyper	Iso/hypo	Hyper	Significant hyper	Significantly appreciable
Mixoma	Significantly hyper	Iso/hypo	Heterogeneous hyper	Variable intensity	Heterogeneous variable intensity
Papillary Fibroelastoma	hypo	Iso/hypo	Iso/hyper	Variable	When appreciable, homogeneous and hyper
Teratoma	hyper	Iso/Hypo	Hyper	Absent uptake	Variable intensity
Lipoma	Hyper with Dark border	hyper	hyper	Absent uptake	Absent uptake

## 4 Technical hints in the pediatric population

As suggested by the literature, the protocol is very similar to adult but adapted to the size and cardiac frequency of the little patient. In detail, spatial and temporal resolution should be increased, balancing them out with noise and acquisition time.

Breath hold acquisitions are more achievable in patients older than 7 years or under general anesthesia, since they can be guided in intubated patients [32]. As an alternative, sedation and/or free-breathing sequences can be an option. Changes in acquisition parameters are needed for free breathing imaging in order to avoid artifacts [32], such as increasing signal averages (to 2–3) and using respiratory navigator sequences triggered at end expiration.

In recent years, innovative approaches to image acquisition and reconstruction are leading toward fast, high-resolution, 3D and even 4D datasets for delineation of cardiac anatomy and function, requiring no or minimal patient cooperation [33]. These techniques all aim at drastically speeding up acquisition time without significantly degrading image quality and mainly consist of acceleration and/or motion correction algorithms. The most renowned is compressed sensing (CS), a technique based on k-space incoherent subsampling, paired with a noise-reduction algorithm employing sparse representation in a nonlinear iterative reconstruction process [34]. Other complementary approaches include radial/spiral sampling and self-navigation techniques. In this context, another vast and thriving topic is that of artificial intelligence (AI), referring to the development of “trained” computerised models to perform tasks typically requiring human intelligence. AI is still in its early stages but has been showing tremendous promise in medical imaging and CMR [35], with new solutions for image acquisition, reconstruction, post-processing and analysis, along with biomarkers for a wide spectrum of cardiac conditions within the field of radiomics. However, the incredible number of AI models and their meaningful integration in clinical routine remains a challenge. Finally, 3D printing-prototyping can be used to produce patient-specifc cardiac models and virtual/augmented reality visualization of cardiac structures, which can prove useful to clinicians and surgeons for preoperative study, practice and communication with patients [36].

## 5 Rhabdomyoma

Rhabdomyomas are the most common primary cardiac tumor in children and infants, commonly appearing during the first year of life with no predilection for cardiac chambers or side of the heart, even if they are most frequently detected in the ventricles [37,38].

They can be intracavitary or intramuscular, with a dimension varying from some millimeters to some centimeters.

In 90% of cases, they are multiple [10]. Association with Tuberous Sclerosis is accurately established. Histopathologically, they are hamartomas composed of enlarged, vacuolated cardiomyocytes. In the majority of cases rhabdomyoma regress spontaneously, without surgical correction.

However, some Rhabdomyomas can be arrhythmogenic and symptoms can include palpitations and syncope. Obstruction of inflow or outflow tract with protrusion of the mass can lead to congestive heart failure.

**Fig. 1:**
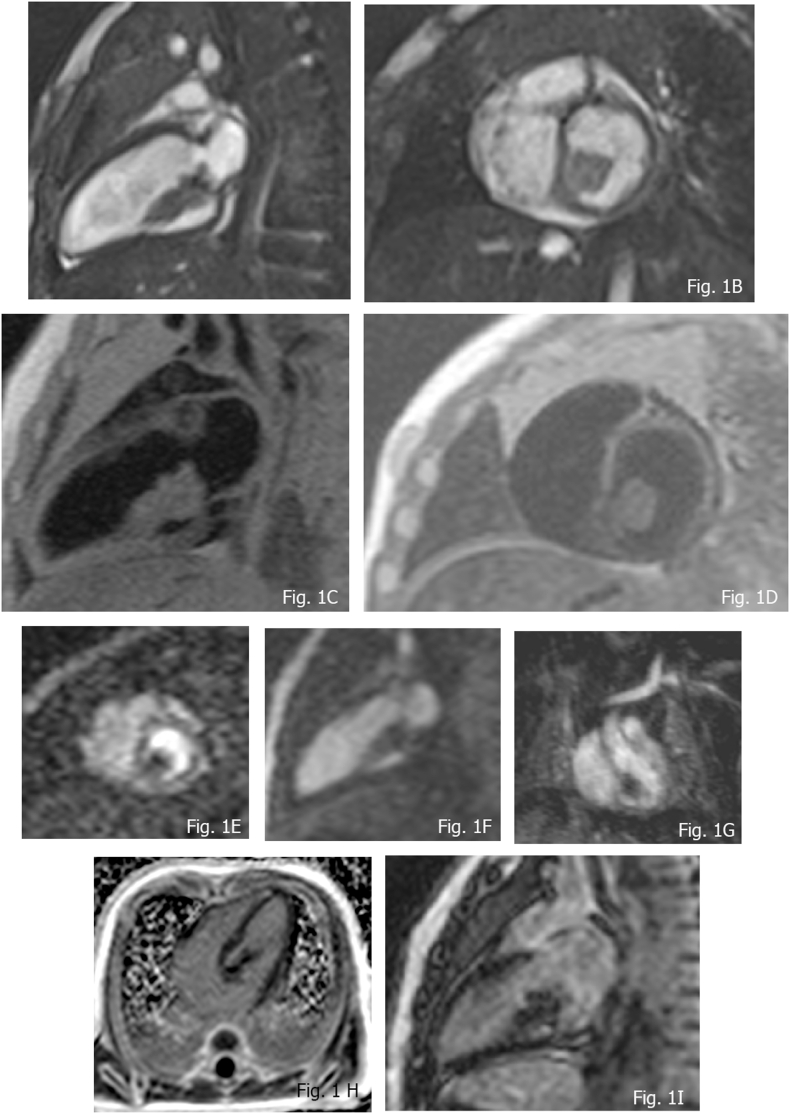
MR imaging of Rhabdomyoma in a 4 months old female. A and B) isointensity at b-SSFP; C) T1w TSE isointensity features; D) isointensity at T2w TSE; E and F) FPP shows absent uptake of the mass; G) Contrast Enhanced MR angiography (CEMRA) detail confirming absent uptake; H and I) isointense presentation at LGE.

CMR aspects: at b-SSFP sequences they appear as iso-hypointense nodules. At T1w TSE they appear isointense compared to the myocardium, while they show variable intensity, commonly described as hyperintense at the T2w [39,40]. LGE is not present or slightly detectable. Low cine b-SSFP and low LGE signal intensity are suggestive, as independent predictor aspects [30] of rhabdomyoma’ (Fig. 1A–I) (see Fig. 1).

## 6 Fibroma

**Fig. 2:**
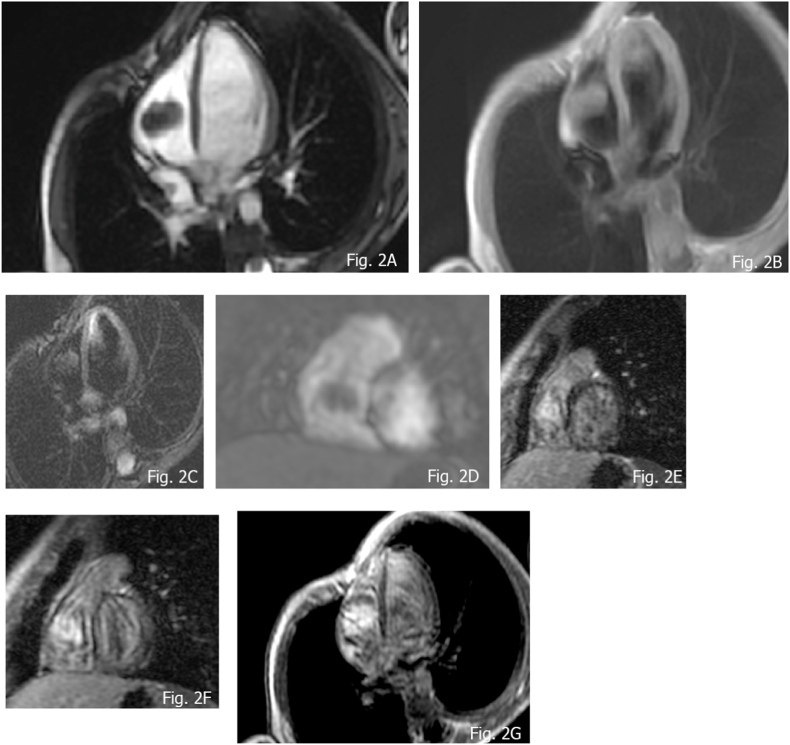
Fibroma in an 8 years old female A) isointensity compare to normal myocardium; B and C) T1 w and T2 w TSE demonstrating isointensity; D) absent uptake of FPP; E) LGE at 7 min showing avid uptake; F) LGE at 11 min, exhibiting persistent significative uptake; G) LGE at 17 min, demonstrating progressive uptake.

**Fig. 3:**
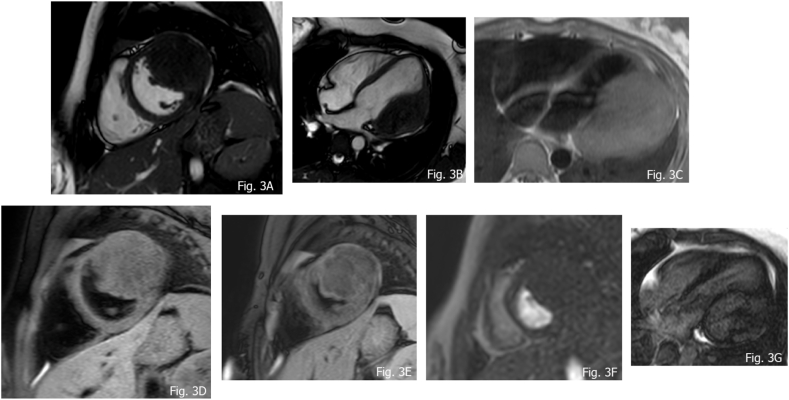
10 years old female, with a large fibroma of the lateral wall of the left ventricle A and B) SSFP showing slight hypointensity; C and D) T1 w TSE demonstrating isointensity appearance; E) T2 SPAIR sequences showing hypointense features; F) FPP exhibiting no uptake at first pass; G) LGE fibroma pattern.

**Fig. 4:**
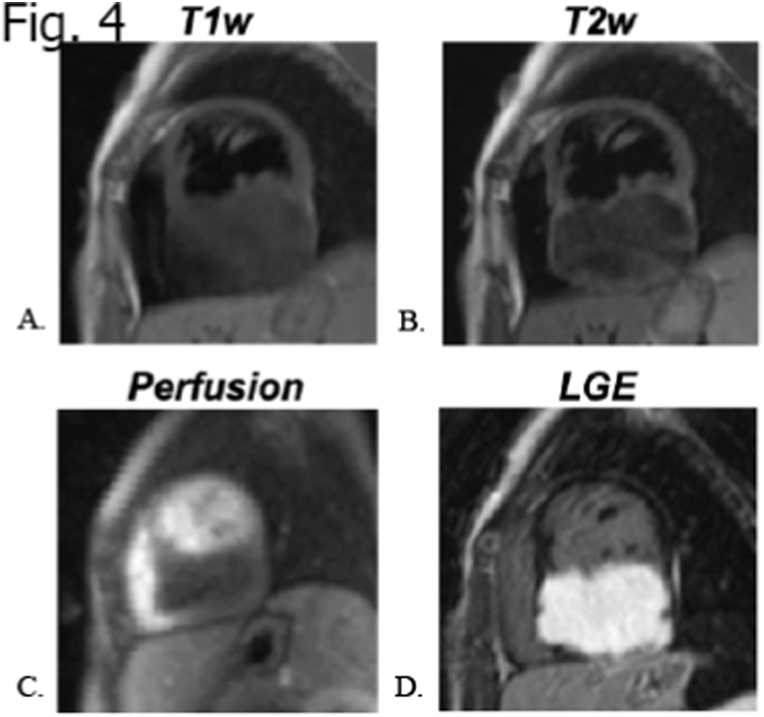
MR study in a 2 years-old girl shows a left ventricular inferior wall mass with mild hypointensity in T1 (A) and T2 weighted (B) sequence, hypoperfusion (C) after contrast administration and marked LGE (D) in keeping with fibromatous tissue.

Fibromas are the second most common cardiac tumor in the pediatric population, representing 12–16% of the primary cardiac tumor in children [41]. Usually solitary and well circumscribed, they arise from connective tissue fibroblasts. They show intramural expansion in the ventricles involving the free wall as well as the septum, sometimes mimicking hypertrophic cardiomyopathy [42]. Fibromas can be asymptomatic in one third of cases; due to the spread in functional myocardium, they can determine congestive heart failure or arrhythmia when involving conducting system. Fibromas do not regress spontaneously and surgery (resection or debulking) is therefore indicated in some settings like underlined by some authors [30,43], specifically when the lesions show one or both of the indicated aspects: 1) hemodynamic impairment; 2) life-threatening arrhythmias because treatment helps to reduce hemodynamic and arrhythmogenic burden. Moreover, left ventricular dysfunction must be considered before and after surgical treatment, as it can be an unknown complication, that can lead to a worse prognosis [44]. Fibromas can show dimension from some millimeters to several centimeters. Calcifications in the core of the mass is a characteristic sign [45,46] and in imaging is appreciated in 25% of cases [46]. Association with Gorlin syndrome and rarely with Familial Adenomatous Polyposis is described in the literature, in this context atrial origin can be observed [10,47]. CMR features of fibromas are well defined borders, generally isointense or hypointense relative to normal myocardium on T1-weighted images and hypointense at T2 weighted images, occasionally they can exhibit some heterogeneous aspects with hyperintensity appearance [31]. Hypointensity is observed at first pass perfusion (FPP), in relation to a weak blood supply. At LGE sequences fibromas tipically show avid, progressive, enhancement with or without central hypoenhancing core. According to several researchers, some independent predictor of fibroma can be acknowledged such as: 1. Larger tumor size (mass-to-cardiac ratio: >0.6) 2. Intramyocardial site; 3. Low Cine SSFP intensity ratio and high LGE signal intensity, with occasionally patchy dark areas [30]. Fibromas can present with some spot calcifications; it might be difficult to recognize those with CMR as calcium characteristically has “no signal”. Therefore, in certain unclear circumstances non-contrast CT can be additionally performed to better characterize the tissue properties of the mass (Fig. 2 A-G, Fig. 3 A-G, Fig. 4 A-D).

## 7 Cardiac Inflammatory Myofibroblastic Tumor

Cardiac inflammatory myofibroblastic tumor (IMT) is a rare mesenchymal neoplasm occurring in infants, children, and occasionally adults, with a prevalence ranging between 0,004 and 0,7% in the world population, regardless of gender and race [48]. As the name suggests, this tumor arises from mesenchymal cells, especially from myofibroblasts [49]. The tumor originates from the inner part of the gastrointestinal tract, airways, central nervous system and endocardium. The word “inflammatory” refers to the histopathological features of IMT, characterized by the presence of immune cells, among which plasma cells, lymphocytes, and eosinophils surrounding elongated myofibroblasts [50]. IMT is known to be a neoplasm of intermediate biologic potential, with 25% of recurrence and 2% of metastasis [51]. As virtually all cardiac tumors, it can be completely asymptomatic or symptomatic, based on the tumor location, size, rate of growth and patient's tolerance. There are no specific signs or symptoms related to cardiac IMT's. However, IMTs are thought to elaborate cytokines that may produce constitutional symptoms and signs such as fever, anorexia, anemia, hyper gamma-globulinemia, leukocytoclastic vasculitis, polyarthritis, and thrombocytosis [52]. The diagnosis of IMT can be challenging due to the wide morphological spectrum exhibited by the tumor. First evaluation usually starts with transthoracic echocardiogram. Furthermore, cardiac magnetic resonance is challenging, allowing tissue characterization by evaluating the intrinsic variable cellular and myxoid percentage composition. The tumor shows a variable expression at T1w and T2w sequences, a slight hyperintensity at cine SSFP, and an isointense or hypointense feature at FPP [30,53]. LGE is variable. The final diagnosis is made by the pathologist after complete surgical resection. Authors have distinguished the histological features of extrapulmonary IMT into three patterns: (a) spindle cells in a myxoid background with a vascular and inflammatory component (myxoid/vascular pattern); (b) compact spindle cells with intermingled inflammatory cells (compact spindle cells pattern); and (c) dense plate-like collagen (hypocellular fibrosis pattern) [54].

**Fig. 5:**
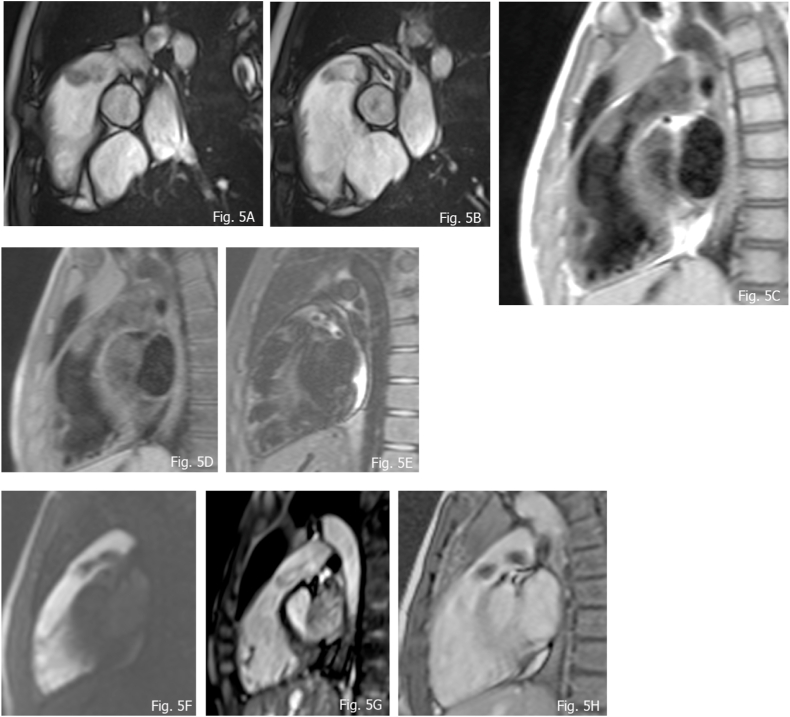
A and B) hyperintense presentation at b-SFFP of a IMT of the RVOT; C) hysointensity at T1 w TSE; D) T1 w FAT SAT; E) mild hyperintensity at T2 w TSE; F) FPP without lesional contrast enhancement; G) 3D WHOLE Heart confirming hypertintensity such as observed in cine b-SFFP; H) absence of delay enhancement. Adapted from *D'Angelo T, Mazziotti S, Inserra MC, De Luca F, Agati S, Magliolo E, Pathan F, Blandino A, Romeo P. Cardiac Inflammatory Myofibroblastic Tumor. Circ Cardiovasc Imaging. 2019 Sep;12(9):e009443.*https://doi.org/10.1161/CIRCIMAGING.119.009443*. Epub 2019 Sep 10. PMID:**31500449**; PMCID: PMC7099846*.

Immunohistochemical analysis is key contributive to the differential diagnosis, objectifying universally positive for vimentin, universally negative for CD34 and generally positive for CD-68 and smooth muscle actin antibodies [55] (Fig. 5A–H).

## 8 Cystic tumor of the atrioventricular node

Cystic tumor of the atrioventricular (AV) node is a rare benign choristomatous tumor, located in the triangle of Koch in the AV nodal region of the atrial septum of the heart [48]. It comprises 2.7% of cardiac tumors, more prevalent in women (with a ratio of 3:1) and is the most common primary cardiac tumor causing sudden death [56].

It is a congenital tumor that arises during embryonic life, from the endodermal layer and ultimobranchial elements. [57,58]; 10% of patients have other midline defects [59]. Histologically, the cysts are benign and lined by flattened cuboidal or squamous epithelium.

Due to these tumor's location, congenital heart block is a typical manifestation and other symptoms include palpitations, dyspnea, syncope dizziness and sudden death [60].

Most atrioventricular nodal tumors are diagnosed incidentally at autopsy, in patients with a history of heart rhythm problems or as the cause of sudden death.

Due to its atypical presentation, Cystic tumor of the AV node is hard to diagnose: the first instrumental approach can be done with the transthoracic echocardiogram, that might show a well-delineated lesion in the area of the AV node [61]. Therefore, CMR is needed for a better diagnosis: CMR shows a cyst in the AV or proximal interatrial septum. The lesion is known to be isointense in T1, Hyperintense in T2 and to have a heterogeneous enhancement at FPP and at LGE [62].

**Fig. 6:**
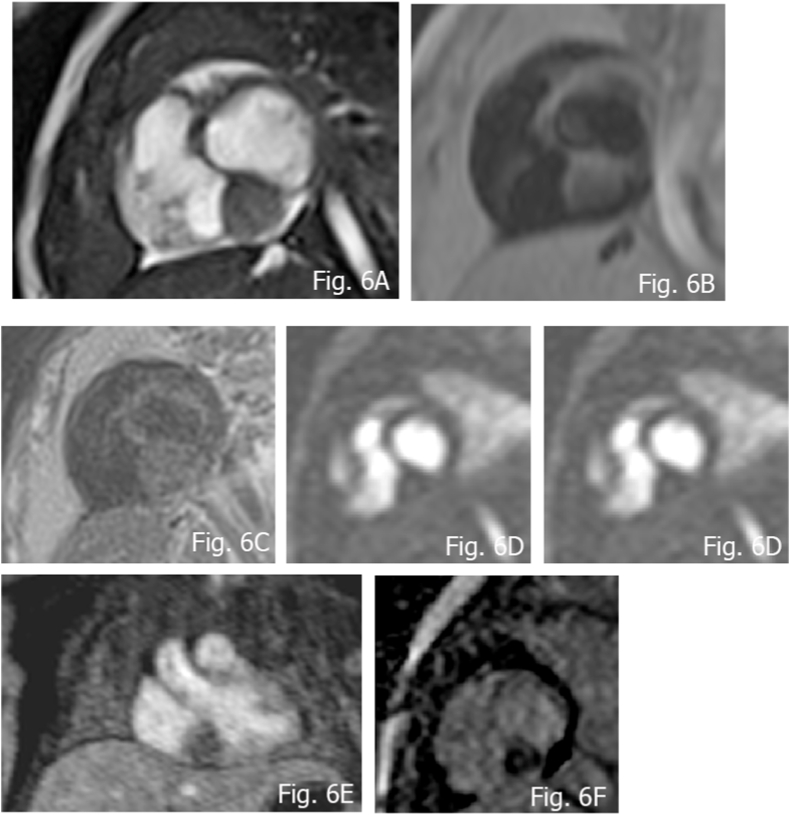
3 months old female with Atypical rhabdomyoma, resembling a cystic tumor of the atrioventricular node. A) Cine b-SSFP nicely delineating the site; B) T1 w FS; C) isointense aspects at T2 Blade FS; D and E) no significant uptake at FPP, as seen at CEMRA; F) absent uptake at LGE.

There are a few case reports of successfully resected atrio-ventricular nodal tumors, as this is the only curative surgery for this kind of tumor [63]. (Fig. 6A–F).

## 9 Hemangioma

Hemangiomas and vascular malformations of the heart are heterogeneous, with a smaller group present at birth or in childhood and a larger group occurring in adulthood [64]. They account for 2,7% of primary cardiac tumors [64] and, in children, the most common location is the right atrium and the left ventricle [65].

Hemangioma has several histological subtypes as: 1) cavernous hemangioma, made of multiple thin- and/or thick-walled dilated vessels, 2) capillary hemangioma, which has lobules of endothelial cells forming small, capillary-like vessels and 3) arteriovenous hemangioma or cirsoid aneurysm that consists of dysplastic thick-walled arterioles, venous-like vessels, and capillaries [66,67].

Cardiac hemangiomas are often clinically insignificant and are mostly diagnosed incidentally. This tumor does not present any specific symptoms and its peculiar manifestation depends on its location, size and speed of growth.

Usually, the first instrumental approach is done with transthoracic echocardiogram, which shows a hyperechogenic lesion. In infants, it is also frequently associated with pericardial perfusion and tamponade [68]. Furthermore, CMR is of fundamental importance in the diagnostic pathway: tumors exhibit a variable and heterogenous signal intensity at T1-and T2-weighted images, with most commonly a hyperintensity appearance at T2W TSE [22]. After gadolinium infusion, rapid homogeneous enhancement is characteristic of capillary hemangioma due to the high vascularity of the tumor, while cavernous hemangioma displays a progressive enhancement. Therefore, it demonstrates high signal intensity at FPP and might vary from isointense or mild hyperintense signal at LGE evaluation [31]. Endolesional calcifications can be present.

Coronary CTA and coronary angiography are also useful to show the distribution of vessels [69].

**Fig. 7:**
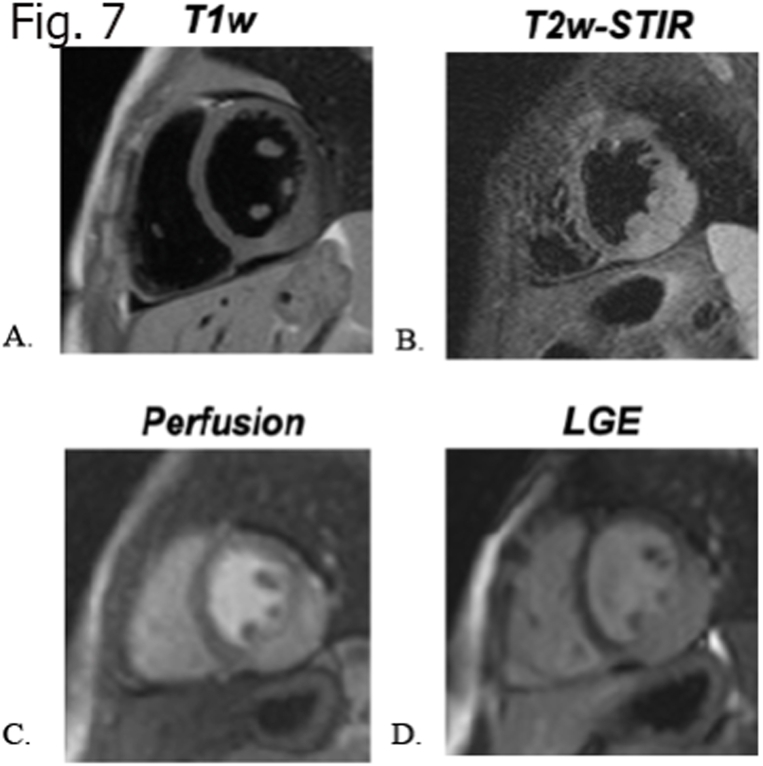
a 6 years old girl with myocardial hemangioma of the left ventricle infero-lateral wall, characterized by mild hypointensity in T1 weighted sequence (A), mild hyperintensity in T2 (B), with hyper-enhancement at first pass perfusion images (C) and mild homogeneous enhancement at LGE (D) sequence with progressive wash-out.

Surgical removal is the choice of treatment for primary cardiac hemangiomas [70]. After complete resection, the prognosis is generally favorable, with a very low recurrence rate. However, even an incomplete resection has been reported to produce long-term survival benefits and improve patient's prognosis and quality of life, by reducing symptoms caused by the tumor [71] (Fig. 7A–D).

## 10 Myxoma

Cardiac myxomas are usually pedunculated and solitary masses, derived from mesenchymal cell precursors [72] and most commonly found in the left atrium, attached by a stalk to the fossa ovalis, but may be appreciated in the right atrium in children [38] Other anatomical origins include the atrial free wall and the mitral valve leaflets; however, these are less frequent [38].

Association with Carney complex (multiple neoplasia and lentiginose syndrome) is well-known in 7% of patients [73].

**Fig. 8:**
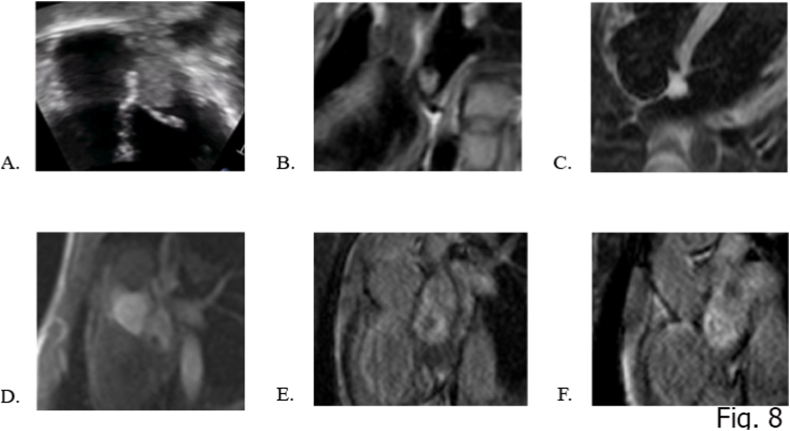
a 15 years-old girl with ectopic beats during exercise test diagnosed with a left atrial sessile mildly hyperechoic mass (A) anchored to the interatrial septum. The MR study shows a rounded mass with high T2 signal (C.) iso-intense in T1w images (B) hypo-enhanced at first pass perfusion (D) with progressive and concentric inhomogeneous LGE (E and F) (Mixoma).

Mean age at diagnosis is 50 years, and approximately 70% occur in women [74]. When occurs in syndromic context, more common in young males, has a multifocal development with multisite origin [75]. Right atrium can be an alternative site as well the mitral and tricuspid valve, and rarely, the ventricles and the pulmonary artery [38]. They can present as sessile or pedunculated mass and are morphologically divided into polypoid and papillary. The polypoid one, when large in size, may present with obstructive symptoms and with a “tumor plop”, occasionally heard on auscultation of the chest. In contrast, papillary myxoma tends to cause embolic events, that can involve every peripheric region. The diagnosis of myxoma in children is very challenging, as this tumor can simulate other, due to the fact that its CMR features resemble the appearance of several cardiac tumors, such as fibroelastoma and malignant tumor [30]. On CMR, a heterogeneous appearance on T1-and T2-weighted images is often found due to the composition of myxomas, which tend to have varying amounts of myxoid, hemorrhagic, ossific, and necrotic tissue [30]. As underlined by Gatti et al. three MRI patterns are evident in black blood sequences: 1) Most frequently it is isointense in T1-weighted and hyperintense in T2-weighted images; 2) it can be hypointense in both T1 and T2 due to the presence of calcifications or 3) extremely hyperintense in T2 with “pseudo-cystic” appearance [25,76]. At first pass perfusion, they exhibit hypointense behavior, with a minimal enhancement compared to myocardium [77]. At LGE, heterogeneous enhancement can be appreciated with areas of iso and hyperintensity presentation. On parametric imaging, myxomas show elevated native T1 and T2 relaxation times and extracellular volume values [78]. As shown in the multicenter study of R.S Bekourim et al. two independent MRI features predictors can be mentioned such as endocardial location and cine SSFP intensity ratio of 1.6 [30]. Resection is required not only for a histological diagnosis but mainly to prevent major complications. Surgical resection is associated with a low operative mortality and good long-term outcome [75]. Annual follow-up with TTE is recommended for a minimum of 4 years because approximately 10%–15% of these tumors recur, most often at the site of the original tumor [78] (Fig. 8A–F).

## 11 Papillary fibroelastoma

Papillary fibroelastomas (PF) are rare in children and often found on the downstream side of valves [79]. Hystologically, they consist of avascular fibroelastic tissue, surrounded by endocardium [80].

They are best visualized on echocardiography and the first sign of this mass is usually embolism [81]. Surgical excision is reserved for large (>1 cm), left-sided tumors, as this dramatically reduces the risk of stroke. Instead, right-sided papillary fibroelastomas are usually managed conservatively unless they are associated with hemodynamically significant obstruction or risk of paradoxical embolism (e.g., in cases of intracardiac shunts) [82].

On T1-weighted imaging on CMR, fibroelastomas demonstrate an isointense or hypointense pattern [30]. On T2-weighted imaging, fibroelastomas show isointense signal compared to normal myocardium when they enclose high fibrous components and hyperintense signal at T2-weighted images in the rest of cases [14,30,83,84].

At b-SSFP sequences, they appear as hypointense, circumscribed, mobile valve nodules with turbulent flow. PFs may have homogeneous LGE [25,83].

## 12 Teratoma

Germ cells tumors are included in the WHO 2015 classification of Heart Tumors, but they are not integrated in the WHO 2021 edition since myocardial germ cells tumors are extremely infrequent and the vast majority develops from the pericardium [85].

Primitive cardiac teratomas are very rare in children: Teratomas in newborns usually occur in the sacrococcygeal area, testis and ovaries and less frequently in the neck or intracranially. Teratomas are embryonal neoplasms including the three germ layers (endoderm, ectoderm, and mesoderm) [85]. Teratoma are usually detected at prenatal evaluation and may lead to fetal demise because of cardiac tamponade and ab-extrinsic compression.

**Fig. 9:**
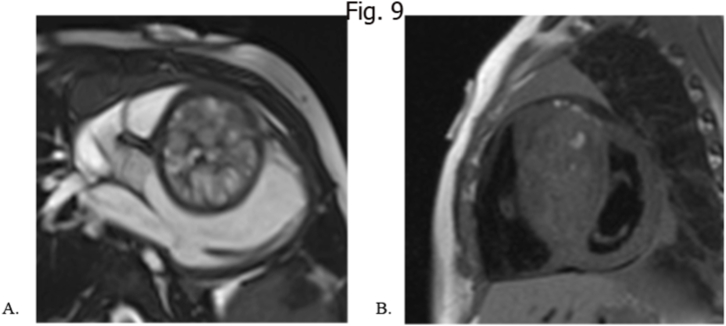
Neonate with large multicystic tumor within the interventricular septum as seen in the balanced SSFP cine images (A) with minimal fat content as visible at the T1w images suggesting a theratomatos intracardiac mass.

T1w and T2w presentation is heterogeneous, with usually hyperintensity at T2w sequences as well as at cine SSFP. They frequently generate compression at superior caval vein, especially in their most common site (right side of the heart) At FFP they show hypointensity and this is crucial for the differential diagnosis with hemangiomas [14,31,86]. Surgical excision is the only effective treatment for cardiac teratoma. Once resected, the prognosis is usually good, as most tumors are benign and require no further treatment (Fig. 9A and B).

## 13 Lipoma

Lipomas are an uncommon type of benign lesions, as they account for 8,4% of primary cardiac tumors [87]. Lipomas are encapsulated and contain adipocytes, usually demonstrating slow growth rate. Multiple lipomas can be associated with Tuberous Sclerosis [88]. Although they can arise from all cardiac chambers and myocardial layers, they are most frequently observed in the left ventricle arising from the endocardial or epicardial layer and they can even arise from the pericardium [89]. Mostly asymptomatic especially in the case of small lesions, they can cause according to their localization, dyspnea, arrhythmia, myocardial ischemia. At CMR, they show hyperintensity at T1 w and T2 w TSE sequences with uniform signal, similar to that of subcutaneous fat. On Fat-saturated sequences they demonstrate typical signal loss within the entire lesion. No LGE is shown as well as no uptake at FFP sequences. B-SSFP can be very useful in order to detect the dark border sign due chemical shift especially in small lesions [87].

## 14 Differential diagnosis

Differential diagnosis is of fundamental importance in the approach to cardiac masses. In order to get the right diagnosis is critical to consider the clinical setting of the patient: symptoms of infection can be suggestive of vegetation rather than a neoplastic lesion. Also, valvular diseases, for example caused by rheumatic fever, can indicate a thrombus rather than a secondary metastasis. Furthermore, it is important to consider the location of the mass: cardiac tumors can develop from every cardiac wall or chamber, but each one has a preferred location. Last but not least, it is essential to consider the mass's characteristics: size, shape, mobility, vascular supply, tissue morphological features and, if necessary, metabolic activity [90]. It is necessary to combine CMR features (especially LGE to detect Fibroma vs Rhabdomyoma and FPP to identify Hemangioma) with the location of the mass, in order to exclude malignancy.

**Malignancy:** it is suspected when is observed pericardial effusion, infiltrative aspects, and elevated T2w signal with strong uptake at FPP are observed. When malignant tumor is suspected, a valid non-invasive diagnostic tool is 18 F-FDG PET [30].

**Fig. 10:**
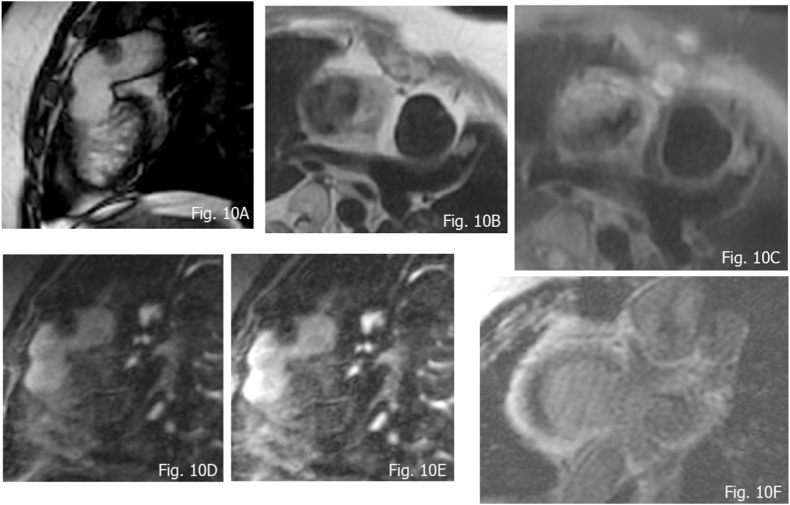
Small subacute thrombus in a 14-year-old female patient with *d*-TGA in the pulmonary artery conduit, revealing hypointensity at b-SFFP (A), an intermediate signal at dark blood (B and C) imaging and hypointensity at FPP (D), without appreciable LGE (E and F).

**Cardiac Thrombus**: In the differential diagnosis apart from malignancy, the presence of thrombus must be considered, since they are the most common intracardiac mass. Thrombus can belong to any cardiac chamber, according to the clinical scenario (for example, in the presence of atrial fibrillation, thrombi are most commonly located in the left appendage and left atrium, or along the right atrium in the presence of central venous catheter etc). Thrombi are non-enhancing masses and, in the vast majority of cases, they show hypointense aspects. Balanced SSFP sequences in clinical practice are extensively used for cine-CMR for the delineation of mural thrombi. However, in some cases the similar signal intensity of contiguous myocardium can fail in the identification. CMR can reveal different signal characteristic depending on the age of the thrombus. Acute thrombus can have intermediate T1 and T2 signal. When subacute, they show a lower signal at T1w sequences due to the methemoglobin and high T2 w signal linked to a higher water content from dissolution of red blood cells [ [39,91]]. Chronic Thrombus appear hypointense at T1 and T2 w sequences for the prevalence of fibrin components. and hypo/iso at the cine SSFP sequences. In relation to avascular composition there is no contrast medium uptake at FPP as well at LGE technique, which, when used with the long inversion times (600 m s), are very useful to detect the nature of thrombus nulling the avascular component and demonstrating hypointensity compared to the myocardium [91]. Rarely, organized thrombus can reveal peripherally enhancement (Fig. 10A–F).

**Fig. 11:**
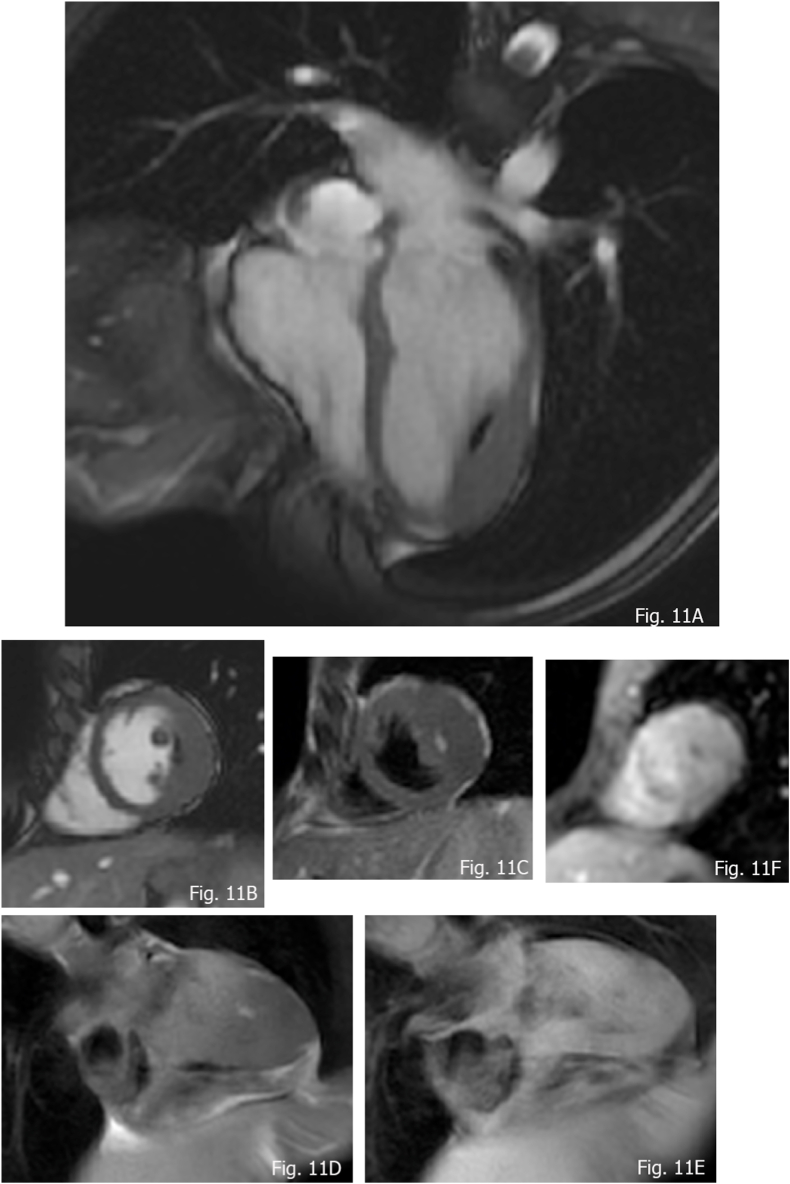
15 years old male with Tuberous Sclerosis and hypertrophic cardiomyopathy. A and B) b-SSFP detecting Indian ink due to chemical shift in small intramyocardial fat deposition; C and D) Double IR T1 revealing high signal; E) Double IR T1 Fs complete signal loss of the lesion; F) no contrast enhancement at FPP.

**Fatty deposits:** intramyocardial fat deposition are described in Tuberous Sclerosis within interventricular septum left ventricle wall, papillary muscle, and even right ventricle. When revealed at MRI, a genetic consultation has to be considered, as a useful tool to complete the diagnosis (Fig. 11A–F). They appear unencapsulated, linear, demonstrating at steady state free precession sequences Indian ink appearance due to the chemical shift. Additionally, they show complete loss of signal on Fat Saturated sequences.

## 15 Conclusions

Primary benign cardiac tumors are very rare in children and the diagnostic and therapeutic pathways reserved to them is usually described by single case reports, leading to the point where a common diagnostic protocol is imperative for the differential diagnosis. MR imaging has a key role in the diagnostic methodology, as it can provide accurate information and details of the mass and can be used as a pre-operative approach. In the next future, the wider availability of compressed sense techniques will allow an easier execution in young non cooperative patients. Moreover, the increased use of the free breathing techniques with motion correction (MOCO) will improve CMR signal quality when scanning young children. Additionally, the future development of artificial intelligence applied in the pediatric CMR field will be an added tool that will permit to gain a more practical approach to the pediatric CMR protocols, helping in the diagnostic algorithm based on a combined association between classic semeiological CMR signs and tissue characterization (multiparametric mapping and LGE) [92]. Finally, in selected complex cases the combination of PET/CMR has a potential role in the differential diagnosis [93].

## Author contribution statement

All authors listed have significantly contributed to the development and the writing of this article.

## Data availability statement

Data included in article/supp. material/referenced in article.

## Additional information

SI - Advanced Imaging in Cardiovascular Diseases.

This item belongs to the item group IG000035.

## Review

All patients (or their relatives/legal guardians) have freely provided written consent for their images to be published and shared for scientific purposes.

## Declaration of competing interest

The authors declare that they have no known competing financial interests or personal relationships that could have appeared to influence the work reported in this paper.
